# Impact of Juvenile Dermatomyositis on Growth, Puberty, Bone Mineral Density, and Body Composition in Children

**DOI:** 10.31138/mjr.280725.ran

**Published:** 2026-03-01

**Authors:** Neha Sudhera, Harvinder Kaur, Vignesh Pandiarajan, Surjit Singh

**Affiliations:** 1 Child Growth and Anthropology Unit, Department of Paediatrics, Advanced Paediatrics Centre, PGIMER, Chandigarh, India;; 2 Allergy & Immunology Unit, Department of Paediatrics, Advanced Paediatrics Centre, PGIMER, Chandigarh, India

**Keywords:** bone density, disease management, immunosuppressive agents, inflammation, lipodystrophy, muscle atrophy

## Abstract

Juvenile Dermatomyositis (JDM) is a rare autoimmune disease characterised by chronic inflammation of the skeletal muscles and skin. This review examines effects of JDM and its treatments, particularly corticosteroids and immunosuppressive therapies, on growth, puberty, bone mineral density (BMD), and body composition. A comprehensive literature search was conducted in PubMed, EMBASE, Scopus, and Web of Science (1992–2025) using combinations of MeSH terms and keywords related to JDM, growth, puberty, BMD, fat distribution, lipodystrophy, and body composition. Methodological quality was appraised using a domain-based checklist adapted from the Newcastle–Ottawa Scale. High-dose corticosteroid therapy, which is commonly used to treat JDM, can lead to growth failure, delayed puberty, and changes in body composition, such as obesity, lipodystrophy, and fluctuations in muscle mass. Furthermore, long-term use of corticosteroids is linked to reduced BMD and an increased risk of osteoporosis and fractures. The chronic inflammation associated with JDM also contributes to metabolic complications, including insulin resistance, hyperlipidaemia, and heightened cardiovascular risk. Studies comparing JDM with other paediatric autoimmune conditions reveal that chronic disease activity and treatment significantly affect growth and pubertal development. However, much of the available evidence is derived from small, heterogeneous cohorts with inconsistent adjustment for factors such as pubertal status, nutrition, physical activity, and disease control. These limitations restrict interpretation and caution against overgeneralisation of findings. Standardised, adequately powered longitudinal studies are needed to clarify developmental trajectories and modifiers of risk. Clinically, proactive multidisciplinary care remains essential to optimise growth, bone health, and long-term metabolic outcomes in JDM.

## INTRODUCTION

Juvenile Dermatomyositis (JDM) is the most common inflammatory myopathy in children, typically diagnosed between the ages of 4 and 10. It is a chronic, rare autoimmune disease that affects multiple systems and is characterised by an immune response against skeletal muscle and other tissues, including the skin, gastrointestinal tract, heart, and lungs.^1,2^ The incidence of JDM is approximately 2.7 to 3.4 cases per million children each year.^3^

Diagnosis criteria were established by Bohan and Peter in 1975,^4,5^ based on the presence of typical skin rash and proximal muscle involvement. The cardinal symptoms of this disease include proximal muscle weakness and distinctive skin changes, such as heliotrope rash on the upper eyelids, Gottron’s papules, and periungual erythema with abnormalities in capillary loops.^6,7^ It may also affect several organs, including the lungs and the digestive tract. In a subset of patients, glucose intolerance, lipodystrophy, and/or calcinosis develop.^8,9^ Recent reviews have explored the clinical, serological, instrumental, and morphological aspects of JDM, along with potential triggers implicated in its pathogenesis.^10^ Standard therapy is currently considered to include glucocorticoids and methotrexate based on a randomised controlled study and expert consensus. Several medications are commonly used in cases of refractory JDM, including azathioprine, ciclosporin, intravenous immune globulins, mycophenolate mofetil, and rituximab.^11^

Chronic inflammatory conditions are often associated with growth failure ranging from slight decrease in height velocity to severe forms of short stature and delays of pubertal onset or slow pubertal progression.^12^ This phenomenon is thought to arise from a combination of factors, including chronic systemic inflammation, nutritional deficiencies, and the side effects of glucocorticoid and immunosuppressive therapy, altered body composition with lean mass reduction and physical inactivity.^13^ This may be through effects on the systemic GH axis that regulates linear growth or through direct effects at the level of the growth plate. Chronic inflammation may lead to a continuum of abnormalities in the systemic GH/IGF-1 axis, including relative GH insufficiency, GH/IGF-1 resistance due to impairment of IGF binding proteins [IGFBPs], down-regulation of GH/IGF receptors and/or impairment of local GH and IGF-1 signalling pathways.^14^ It also suppresses the hypothalamic-pituitary-gonadal axis, which can delay puberty by affecting the secretion of gonadotropins (LH, FSH), and sex hormones (oestrogen, testosterone). Furthermore, growth suppression may be exacerbated by underlying disease activity and the timing of corticosteroid initiation, with those receiving early and prolonged treatment during periods of rapid growth being more affected.^15^ Immunosuppressive drugs, such as methotrexate, azathioprine, and biologics like rituximab, may mitigate the side effects of corticosteroids but still have potential impacts on growth and puberty.^16^

A common adverse effect of long-term corticosteroid and immunosuppressive therapy is reduced BMD, resulting from multiple mechanisms. These include decreased intestinal calcium absorption, increased renal calcium excretion at the distal tubule, suppression of osteoblast activity with promotion of apoptosis, inhibition of local IGF-I and IGFBP production essential for bone metabolism, and reduced osteocalcin synthesis. Long term treatment with corticosteroids is associated with osteoporosis and vertebral fractures.^17^ Studies suggest that the reduced bone mass observed in JDM children is a multifactorial process, with certain aspects of JDM contributing to this condition.^18,19^ Consequently, JDM patients tend to develop osteoporosis in adulthood due to low bone reserves acquired during adolescence and early adulthood.^19^

A well-recognised adverse effect of corticosteroid therapy is fat redistribution, characterised by central adiposity with features such as moon face and buffalo hump. In JDM, prolonged steroid exposure contributes to increased body fat and reduced lean mass, predisposing patients to obesity,^20^ dyslipidaemia,^21,22^ premature atherosclerosis,^23^ and skin changes associated with excess cortisol.^9^ In addition, chronic inflammation in JDM contributes to metabolic alterations that heighten the risk of obesity.^23,25^ Persistent skeletal muscle inflammation a defining feature of the disease not only compromises muscle strength and mobility but also adversely impacts the patients’ overall metabolic profile. Corticosteroids enhance lipogenesis and disrupt fat distribution, leading to characteristic physical features and changes in body composition. Methotrexate and Azathioprine have a less pronounced impact on body composition compared to corticosteroids, but some studies have shown that they still contribute to increased fat mass, particularly when combined with corticosteroids.^26^

Among autoimmune diseases, JDM is one of the better-recognised conditions associated with lipodystrophy (LD), characterised by localised or generalised loss of subcutaneous fat, often accompanied by metabolic complications such as insulin resistance (IR) and hyperlipidaemia.^27^ Several studies have estimated the prevalence of lipodystrophy in JDM to range from 10% to 40%.^28–31^ Reported risk factors include a chronic disease course, panniculitis, facial rash, calcinosis, joint contractures, muscle atrophy, and positivity for anti-TIF1-γ antibodies.^32,33^

JDM poses a unique challenge in paediatric care because it not only disrupts normal immune function but also impairs growth and development. The chronic inflammation associated with JDM, compounded by treatments like corticosteroids and immunosuppressive drugs, significantly impacts bone mineral density, growth, puberty, and body composition. This review summarises the current understanding of how JDM and its treatment modalities affect bone mineral density, growth, puberty, subcutaneous fat distribution and body composition.

## SEARCH METHODS

**Figure 1.**
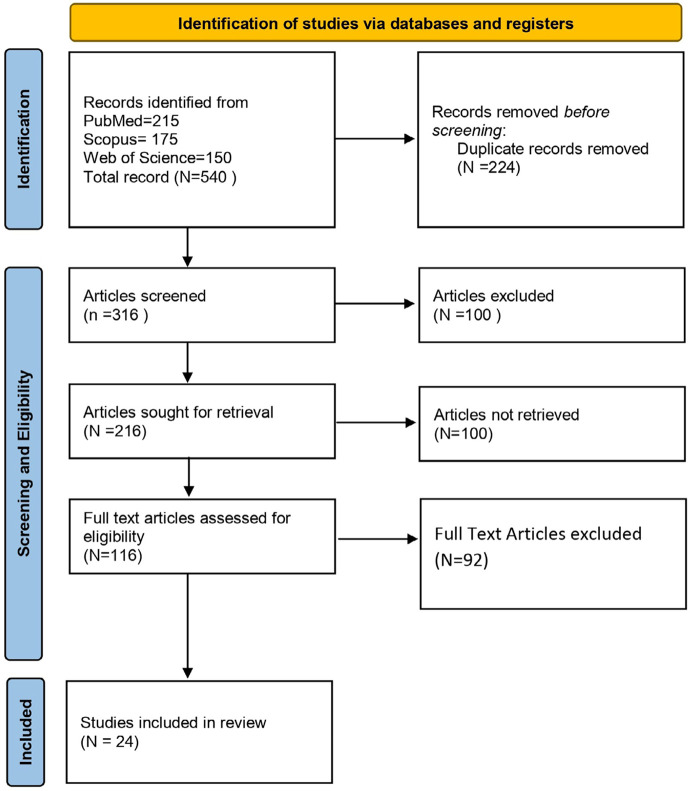
Identification of studies via databases and registers.

A comprehensive literature search of PubMed, EM-BASE, Scopus, and Web of Science was conducted to identify studies on juvenile dermatomyositis (JDM) in paediatric populations, with particular emphasis on publications from 1992 to 2025. The search strategy utilised combinations of keywords and MeSH terms such as “juvenile dermatomyositis,” “body composition,” “bone mineral density,” “growth,” “puberty,” “lipodystrophy,” “bioelectrical impedance analysis,” “phase angle,” and “DXA” in conjunction with Boolean operators. In addition, reference lists of relevant articles and systematic reviews were screened to identify further eligible studies. Studies were included if they involved paediatric patients (<18 years) with JDM, provided original clinical or observational data on bone mineral density, growth, puberty, fat distribution, lipodystrophy, or body composition, and employed validated assessment methods. Only articles published in English were considered. Eligible study designs comprised prospective and retrospective cohort studies, cross-sectional studies, and systematic reviews. Exclusion criteria encompassed case reports with fewer than three patients, conference abstracts without full text, animal studies, studies on adult dermatomyositis, commentaries, opinion pieces, editorials, and studies lacking relevant outcomes. No restrictions on geographic setting were imposed. Two reviewers independently screened the titles and abstracts, followed by full-text assessments. Disagreements were resolved through consensus or, when necessary, by consultation with a third reviewer. Data extraction focused on study characteristics, patient demographics, disease features, assessment methods, and key outcomes. The methodological quality of included observational studies was evaluated using a domain-based checklist adapted from the Newcastle–Ottawa Scale, assessing selection, measurement validity, confounding control, and completeness of reporting. Each domain was rated as low, unclear, or high risk of bias, and these evaluations were integrated into the narrative synthesis, with greater weight assigned to findings from studies at low risk of bias (**Supplementary Table**). The search yielded 500 articles, with 316 selected for pre-screening after removal of duplicates. Reviewers H.K. and N.K. independently screened the resulting articles and abstracts according to the inclusion criteria. Ultimately, 116 articles addressing the impact of JDM and its therapies were identified. After full-text review for relevance, 24 articles met the inclusion criteria and were incorporated into the final review (**Figure 1, Table 1**)

**Table 1.** Summary of the studies included in the review.

**Category**	**Study (Country)**	**n (JDM / Control)**	**Mean/Median Age (years)**	**Duration of disease (years)**	**Main Measures**	**Key Findings**
**Bone Health**	Alsufyani^43^ CANADA	7 / –	11.4 ± 2.9	2.9	BMD (Total Body, Lumbar Spine [LS], Hip), Tanner staging	27% had ↓BMD z-score; associated with ↑steroid dose, younger age, prepubertal; no consistent predictors.
	Castro^19^ BRAZIL	10 / 20	11.8 ± 3.2	3.6	LS, BMD, Tanner staging	7/10 osteopenia; LS BMD ↓ vs Controls; weight ↔ bone mass; no link with steroids/duration.
	Falcini^45^ ITALY	11 / 55	13.0 ± 2.7	6.4	LS, BMD, Broadband Ultrasound Attenuation (BUA) by calcaneal US	↓BMD & BUA in JDM vs Controls.
	Santiago^24^ BRAZIL	20F / 20	13.5 ± 4.2	3.9	BMD of Femoral Neck (FN), Total Femur (TF), Whole Body (WB), BMAD, Lean Mass (LM), Fat Mass (FM)	↓BMAD in FN, TF, WB (p<0.005); ↓LM and no difference in FM vs controls; 60% had <25th height percentile.
	Rouster-Stevens^67^ USA	37 / 44	6.3 ± 2.4	0.9	LS BMD, muscle-derived enzymes, RANKL and OPG enzymes.	61% LS BMD z < −1.5; ↑RANKL:OPG ratio in low BMD group.
	Marstein^46^ NORWAY	59 / 59	15.3	6.5	BMD (WB, LS, hip, forearm)	25% fractures; ↓BMD in spine/WB vs controls, more severe in <20y.
	Stewart^18^ CANADA	15 / –	7.9	–	LS BMD	Majority osteopenic, incl. pts in remission.
**Growth & Puberty**	Aikawa^37^ BRAZIL	12 / 24	Onset: 10	8.2	FSH, LH, sex steroids, menstruation history	Menarche delayed (13 vs 11 y); ↓ovarian reserve; normal cycles as compared to controls.
	Nordal^34^ Multinational	196 / –	8.9	0.06	Height z score, puberty	Height deflection in 25% F / 31% M; growth failure 15–21%; delayed puberty ∼36%.
	Soni^38^ INDIA	35 / –	–	–	Tanner staging	Menarche delayed ∼1 y; boys’ G2 delayed 6 mo vs local peers.
	Ravelli^35^ Multinational	490 / –	6.9	7.7	Growth, puberty	Growth failure 8%, lipodystrophy 9.7%, osteoporosis 6%.
	Soni^39^ INDIA	35 / –	–	–	Height, weight, skinfolds	Boys taller but lighter & ↓fat vs girls; catch-up growth in girls >12 y.
**Body Composition & Lipodystrophy**	Coyle^8^ USA	16 / –	–	3.2	BMI, MRI, IR	LD 17.6%; MetS 25%; IR ↔ MRI muscle damage.
	Huemer^28^ CANADA	20 / –	11.8 ± 5.6	2.0	Lipids, OGTT	LD 25%; IR 50%.
	Khojah^9^ USA	68 / –	7.0 ± 3.4	2.9	DXA	LD 29%; 19% fractures; ↓BMI%, LM loss despite high FM%.
	Sharma^56^ INDIA	37 / –	13.1	6.1 y	MMT8, CMAS, CAT	LD 32%.
	Pugliese^51^ BRAZIL	30 / 24	11.8 ± 4.0	3.3 y	BIA, skinfolds	Obesity 36.7%; very high BF% 66.7%; ↓PhA linked to severe disease & shorter duration.
	Verma^31^ INDIA	20 / 20	Boys: 11.5 ±3.7; Girls: 8.0 ±3.6	–	Height, skinfolds	↓Wt, ↓Ht vs Controls; most affected sites: midaxillary (65%), suprailiac (60%).
	Witczak^52^ NORWAY	59 / 59	21.5	7.9 y	DXA, hs-CRP	↓LM, ↑BF%, ↑central adiposity; assoc. with inflammation & muscle weakness.
**Metabolic & CV Risk**	Kozu^21^ BRAZIL	25 / 25	11.5 ± 3.8	–	Lipids, ESR, CRP	Dyslipidaemia 36%; ↑TG, ↓HDL; associated with ↑inflammation & cyclosporine use.
	Wahezi^23^ USA	20 / 20	12.1 ± 4.4	–	Lipids, BP, FMD	Obesity 40%; BMI ↔ better endothelial function in JDM only.
	Pereira^20^ BRAZIL	20 JDM (from JIA/JSLE cohort)	12.5 ± 4.1	4.5 y	BMI, waist/height, diet, lipids	Dyslipidaemia in 83%; parent–child link in fat intake & TGs.
	Bingham^32^ USA	353 / –	17.1	–	MRI, DXA, metabolic parameters	LD 7.9%; 38% glucose intolerance; 47% ↑LDL; 60% ↓HDL; 57% menstrual irregularities.
	Sanner^36^ NORWAY	60 / –	9.0	16.8 y	DAS, MDI	LD 17%; growth failure 10%; irregular menses 20%.

All patients received Corticosteroids/methotrexate or DMARDS.

## IMPACT OF JUVENILE DERMATOMYOSITIS ON GROWTH AND PUBERTY

Evidence on growth and pubertal outcomes in JDM arises from heterogeneous cohorts varying in age, disease duration, treatment exposure, and pubertal stage at onset, which limits direct comparability. The most consistent finding is that high-dose corticosteroid therapy during active disease is associated with transient reductions in height velocity and delayed pubertal milestones, with some recovery over time. In a multinational cohort^34^ of 196 JDM patients (Nordal et al., 2020) treated with corticosteroids, growth failure was present at baseline in 16.5% of females and 9.6% of males. Predicted parent-adjusted height fell significantly during the first year, but recovered within two years, indicating catch-up growth. Despite this recovery, height deflection at final follow-up was seen in 25% of females and 31% of males. Delayed pubertal onset was observed in 18% of patients with breast stage 2 (B2) occurring after age 13 years in girls and Genitalia stage 2 (G2) occurring after age 14 years in boys and overall, 36.4% of girls and 35.5% of boys had a delay in pubertal onset, pubertal tempo, or menarche at >15 years (in girls). Notably, cumulative glucocorticoid dose was not a significant predictor in this study, suggesting that timing of exposure relative to puberty may be more influential. Other cohorts (Verma et al.; Huemer et al.)^28,31^ similarly reported challenges in attaining height and weight milestones, reinforcing the treatment-related impact.

In contrast, Ravelli et al.^35^ and Sanner et al.36 found chronic or prolonged disease activity not steroid exposure to be the strongest predictor of long-term growth failure, highlighting the role of persistent inflammation. Reproductive outcomes also vary across cohorts. Aikawa et al.^37^ found that girls with JDM had low follicular reserves, regular menstrual cycles, but delayed menarche and lower progesterone levels, and suggested possible subclinical corpus luteum dysfunction associated with this condition. In contrast, Soni et al.^38^ observed comparable pubertal attainments in boys with JDM and only slightly delayed menarche in girls compared to healthy controls, with the age of B2 and menarche onset similar to other JDM peers. In their another study on the physical growth, JDM children were lighter and shorter than controls, but girls showed catch-up growth—becoming heavier and taller after ages 12 and 14, respectively.^39^ Across studies, consistent reporting of growth and puberty outcomes stratified by pubertal status, nutritional intake, physical activity, disease activity indices, and cumulative steroid dose would improve comparability and clarify the relative contributions of treatment versus disease in growth and pubertal impairment.

## BONE HEALTH IN JUVENILE DERMATOMYOSITIS

Data on bone metabolism in children with juvenile dermatomyositis (JDM) are limited as compared to adults,^40^ but paediatric rheumatic diseases are known to increase fracture risk.^41,42^ BMD outcomes in JDM also derive from heterogeneous samples differing in age, disease course, glucocorticoid exposure, and imaging methodology. Osteoporosis affects approximately 6% of JDM patients worldwide.^35^ In this review, bone density findings are interpreted according to paediatric densitometry principles outlined in the Paediatric DXA Interpretation box.

The most robust signal is that high-dose or prolonged corticosteroid therapy during active disease is linked to persistent osteopenia, as shown by Stewart et al.,^18^ who reported deficits persisting up to eight years post-steroid withdrawal. Alsufyani et al.^43^ demonstrated that cumulative glucocorticoid dose, but not disease activity, was associated with lower BMD across multiple paediatric rheumatic diseases, including JDM. Perez et al.^44^ also reported marked bone mineral loss and reduced calcium absorption during acute steroid-treated illness. Falcini et al.^45^ noted reduced lumbar spine BMD by DXA and lower calcaneal ultrasound values; the latter should be interpreted cautiously given its paediatric limitations.

Conversely, some cohorts implicate inflammation rather than treatment. Santiago et al.^24^ reported reduced size-adjusted volumetric BMD (vBMD) in the femoral neck, total femur, and whole body, alongside lower, though still normal, serum calcium. Marstein et al.^46^ found age-specific patterns: patients <20 y had lower BMD Z-scores correlating negatively with prednisolone dose, while those >20 y had forearm bone loss linked to inflammatory markers. The chemokine IP-10, a biomarker of JDM activity, was inversely associated with BMD Z-scores in both age groups.

Contradictory findings, such as those of Castro et al.,^19^ who found reduced lumbar spine BMD without association to steroids or disease parameters, may be explained by differences in measurement sites, Z-score adjustments, sample sizes, and pubertal status distributions. Not all cohorts reported deficits as Chavelier et al.^47^ observed no osteopenia or osteoporosis—again underlining the need for standardised, stratified reporting. Given that both treatment and inflammation can impair bone accrual, BMD assessment at diagnosis (before starting steroids) and periodic monitoring are warranted, with early interventions to minimise steroid burden, ensure adequate vitamin D/calcium intake, promote weight-bearing activity, and address active inflammation.

## PAEDIATRIC DXA INTERPRETATION - KEY POINTS

When interpreting bone densitometry in children, several pediatric-specific considerations are essential. Smaller bones in children can yield artificially low areal bone mineral density (aBMD) on DXA so Z-scores should be adjusted for height or bone size to avoid underestimating bone health. Lumbar spine (L1–L4) and total body less head (TBLH) are recommended for children; femoral neck may be useful in older adolescents. Bone Mineral Apparent Density (BMAD) adjusts aBMD for bone size and geometry, improving accuracy in growing children. Particularly useful in those with delayed growth, chronic illness, or skeletal disproportion. In pediatrics, Z-scores (age- and sex-matched) are used instead of T-scores. A Z-score ≤ −2.0 is considered “low bone mass for age”, but must be interpreted alongside clinical context (fracture history, underlying disease). Always specify skeletal site, adjustment method, and reference databases used to aid comparability across studies. Calcaneal ultrasound is radiation-free and portable but measures only one peripheral site, mainly trabecular bone, and lacks robust pediatric reference data—results are not directly interchangeable with DXA.

## OBESITY AND METABOLIC CHANGES IN JDM

Studies on obesity in JDM vary in sample size, age at onset, and steroid exposure, complicating synthesis. Pereira et al.^20^ conducted a study to examine a possible association between food consumption, BMI, and biomarkers related to cardiovascular risk in children and adolescents with chronic rheumatic diseases and those of their parents. They found that 80% of over-weight children/ adolescents also had parents with the same diagnosis. Patwardhan et al.^48^ assessed JDM patients who were overweight or obese at diagnosis (BMI > 85th percentile) and noted that obesity correlated with a more severe and prolonged disease course, along with complications such as calcinosis. Khojah et al.^9^ evaluated body composition using DXA in JDM patients undergoing glucocorticoid therapy, noting increased weight gain and total body fat mass, with 30% of subjects reaching the 98th percentile BMI for their age. While long-term glucocorticoid therapy is linked to weight gain, Wahezi et al.^23^ observed potential benefits of glucocorticoids in overweight and obese JDM patients, such as better-preserved vascular endothelium, which may reduce atherosclerosis and cardiovascular risk. A cross-sectional study involving 17 patients with severe JDM identified a high prevalence of overweight and obesity, alongside metabolic abnormalities including hypertriglyceridemia and insulin resistance.^8^

In JDM, adipokines and adipose tissue contribute to an inflammatory environment due to altered pro-inflammatory cytokine release.^49^ It seems that the predisposition to obesity in JDM patients is likely multifactorial in a setting of chronic inflammation, therefore it would be preferable to undertake some strategies which would improve overall health. Thus, addressing obesity in JDM requires a comprehensive approach, including dietary modifications, exercise programs, and strategies to reduce glucocorticoid use where possible.

## BODY COMPOSITION AND MUSCLE DYSFUNCTION IN JDM

Research using DXA, BIA, and MRI indicates that active disease and inflammation are associated with lower muscle mass and higher fat mass in JDM, though results vary between studies, possibly due to differences in disease duration, treatment phase, and assessment method. **Table 2** summarises the strengths and limitations of commonly used modalities. Bioelectrical impedance analysis (BIA) is a validated method for assessing body composition. Phase angle (PhA), a key impedance parameter, reflects cellular membrane integrity and fluid balance and is linked to clinical prognosis in various diseases. Research indicates that PhA correlates with lean muscle mass and disease activity, serving as a marker of cellular function and nutritional status.^50^ Pugliese et al.51 have shown that JDM patients with higher PhA values exhibit better muscle mass and nutritional status. This correlation may be explained by the disease course as shorter disease duration was linked to more severe disease manifestations and lower PhA values. However, BIA and PhA measurements can be influenced by hydration status, recent physical activity, and device-specific algorithms. Standardised measurement protocols and paediatric-specific reference data are essential for reliable interpretation in JDM. Additionally, research by Witczak et al.^52^ in 2022 examined the distribution of muscle and fat mass assessed by DXA in patients with long-standing JDM compared to age- and sex-matched controls. Lower muscle mass and higher body fat percentage was associated with active disease, inflammation, and muscle weakness. While one study reported lower skeletal muscle mass in female patients compared to controls,^24^ other studies found no differences in body fat between patients and controls.^21,24^ These findings indicate that consistently high levels of pro-inflammatory cytokines may play a role in skeletal muscle damage in JDM.^53,54^ This connection is particularly important for patients, as it links higher body fat and inflammation to cardiovascular disease markers.^55^ Understanding these relationships can help address metabolic issues like adiposity, hyperinsulinemia, insulin resistance, elevated triglycerides, and low HDL cholesterol, paving the way for better health outcomes. Discrepancies, such as the absence of fat mass differences in some cohorts, may reflect methodological variability or small sample sizes. Standardising body composition assessment protocols and reporting by disease activity, pubertal status, and treatment history would help resolve these inconsistencies.

**Table 2.** Comparative overview of body composition assessment techniques in Juvenile Dermatomyositis (JDM).

	**Advantages**	**Limitations**	**Clinical Relevance in JDM**
**DXA**	Gold standard for total and regional fat, lean mass, and bone mineral density; reproducible; relatively quick	Low radiation exposure; limited portability; cost; may underestimate fat in very lean patients	Detects muscle wasting, fat redistribution, and bone density loss in a single scan
**BIA**	Portable, inexpensive, quick (<1 min); no radiation; can estimate phase angle (cellular health marker)	Influenced by hydration, recent meals, physical activity, and device algorithms; less accurate for regional composition	Useful for routine monitoring in clinics; PhA correlates with muscle quality, function, and disease activity
**MRI**	High-resolution imaging of regional fat and muscle; no radiation; gold standard for muscle infiltration/fat replacement	Expensive; time-consuming; limited availability; requires patient cooperation (motion artifacts)	Useful for detailed evaluation of muscle pathology and fat infiltration in research or complex cases

## LIPODYSTROPHY IN JDM

Lipodystrophy is a late complication of JDM and is associated with more severe, chronic disease and other sequelae such as calcinosis, insulin resistance, overt diabetes, and hypertriglyceridemia. Lipodystrophy prevalence in JDM varies widely (20–65%) across studies, reflecting differences in definition, detection methods, and disease chronicity. It is more frequent in patients with severe or prolonged disease and can present as generalised, partial, or focal fat loss, sometimes with compensatory fat deposition. In a study by Verma et al.^31^ it was found that 65% of patients with JDM exhibited a loss of subcutaneous fat when quantified by skinfold thicknesses, compared to only 40% based on physical appearance alone. The sub-cutaneous fat loss may be related to the release of inflammatory mediators and cytokines associated with the disease. Similarly, Sharma et al. reported that 21.6% of patients showed lipodystrophy as a sign of cutaneous damage assessed by abbreviated Cutaneous Assessment Tool (aCAT).^56^

**Figure 2.**
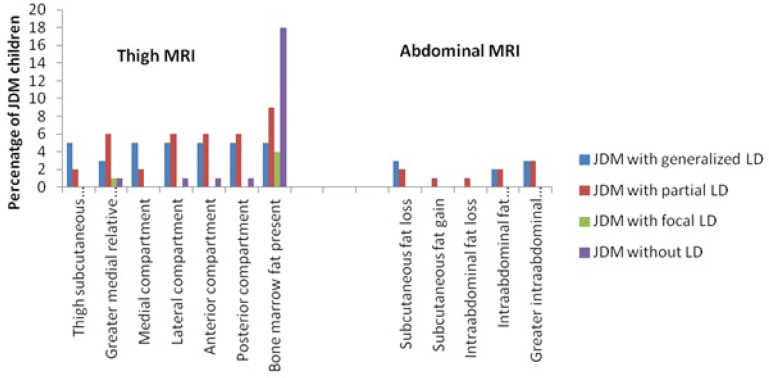
MRI of different compartments.

In a study conducted by Bingham et al.,^32^ among 29 patients with lipodystrophy identified on the basis of physician assessment, 8 had generalised LD, 16 had partial LD, and 5 had focal LA. MRIs were obtained to assess muscle disease or to determine areas of fat loss. Patients with generalised LD invariably had fat loss from all extremities and buccal fat loss, as well as fat loss from the abdomen and trunk. Although the majority of patients with partial LD had fat loss from the lower extremities, others had increased fat deposition. Patients with focal LA had fat loss in the buttocks (in 2 of 5 focal LA patients) or thigh, at a site of calcinosis. MRI showed subcutaneous fat loss in both the thighs and the abdomen. Fat redistribution, with increased fat deposition in certain areas, was a common feature in the generalised (3 of 8) and partial (11 of 16) LD patients, but was not observed in focal LA patients (**Figure 2**).

It was also found that some had a normal total percentage of body fat as measured by dual-energy x-ray absorptiometry (DXA). This might seem inconsistent with a diagnosis of lipodystrophy but can be explained by observed fat redistribution. Long-term corticosteroid use in these JDM patients, especially those with long-standing active disease, may also contribute to a higher total body fat content despite localised fat losses.^57^ A recent retrospective study by Kapetanović et al., 2025 found that lipodystrophy was found in 22.7% of JDM patients based on the clinical inspection of the patients.^58^ Therefore, it is essential to monitor JDM patients closely for the development of lipodystrophy and associated metabolic abnormalities, particularly several years after the onset of JDM in cases with ongoing disease activity.

## COMPARISON WITH OTHER RHEUMATIC DISEASES

Rheumatic diseases, particularly juvenile-onset systemic lupus erythematosus (JSLE), can significantly affect growth, puberty, and body composition in children and adolescents. Several studies highlight the impact of this disease on pubertal development, with Rygg et al.^59^ reporting delayed puberty in 15.3% of females and 24% of males with JSLE. Additionally, 36.1% of females and 44% of males exhibited some degree of delayed pubertal development, while 21.9% of females experienced delayed or absent menarche. Nori et al.^60^ found that SLE girls, were shorter and lighter than their healthy peers, with 18.4% being short-statured and 18.3% showing growth failure. Notably, younger age at diagnosis and longer therapy duration were associated with delayed breast development and menarche in these patients. These findings suggest a direct link between disease activity and pubertal delay in SLE patients. In a study by Taman et al.^61^ it was found that 47.6% JSLE and 17.4% JIA girls had menstrual abnormalities with SLICC/ACR damage index statistically higher in JSLE with abnormal menstrual cycles.

In addition to growth and puberty, BMD is frequently compromised in rheumatic diseases. Warady et al.^62^ demonstrated that children with rheumatic conditions who received adequate calcium and vitamin D supplementation showed significant improvement in spinal bone density. Conversely, Reed et al.^63^ found no significant increase in BMD in children with juvenile rheumatoid arthritis after vitamin D supplementation. Corticosteroids, often used in the management of rheumatic diseases, have been shown to negatively affect bone health. Studies by Haugeberg et al.^64^ and Trapani et al.^65^ revealed significant reductions in bone mass, particularly in the femoral neck and hip, with cumulative corticosteroid use being inversely correlated with BMD. Interestingly, Castro et al.^19^ did not find a significant correlation between cumulative corticosteroid dose and reduced bone mass in JSLE patients, indicating that other factors may contribute to bone density changes. Additionally, research by Lilleby et al.^66^ in 68 SLE patients found significant changes in body composition, including higher fat mass and lower lean mass, with corticosteroid use being a major contributing factor. These cumulative studies underscore the complex interactions between disease activity, treatment, and the development of children with rheumatic diseases, highlighting the need for careful monitoring and management of growth, puberty, and bone health.

Earlier research in JDM has largely concentrated on clinical, serological, and morphological characteristics of this disease. This review for the first time brings together evidence on growth, puberty, bone health, and body composition outcomes in JDM, highlighting how these are shaped by disease activity, glucocorticoids treatment, and modifiable lifestyle influences.

## CONCLUSIONS

Juvenile dermatomyositis has wide-ranging impacts on growth, puberty, bone health, and body composition, driven by the combined influences of chronic inflammation, glucocorticoid exposure, nutritional status, and physical activity levels. Delayed puberty and reduced growth velocity are common, with catch-up growth often incomplete when high-dose or prolonged steroid therapy coincides with critical growth periods. Bone mineral accrual can be compromised from early in the disease, most consistently in patients with high cumulative steroid exposure, though delayed puberty, suboptimal calcium/vitamin D intake, and ongoing disease activity also play important roles. Alterations in body composition, including reduced lean mass, increased adiposity, and, in some cases, lipodystrophy, may arise from both disease and treatment, with the latter phenotype carrying early metabolic risks such as insulin resistance and dyslipidaemia. Obesity, whether steroid-induced or lifestyle-related, further amplifies cardiovascular and musculoskeletal risk. Interpretation of the current literature is limited by heterogeneous study cohorts, variable definitions, and insufficient stratification by pubertal status, nutrition, physical activity, and disease control, emphasising the need for standardised longitudinal research. Clinically, proactive multidisciplinary care with growth and bone health surveillance, GC-sparing approaches, metabolic risk screening, and targeted nutrition and rehabilitation remains essential to optimise long-term outcomes.

## DECLARATION

No part of this review is copied, submitted, or published elsewhere in whole or in part in any languages. All authors take full responsibility for the integrity and accuracy of all aspects of the work.

## CLINICAL TAKE-AWAY

Baseline anthropometry and after every 3–6 months; body composition analysis every 12–24 months. Identify delayed puberty (B2 > 12 y, G2 > 14 y) and if needed a check by endocrinologist. Correct nutritional deficits (energy, protein, Ca, Vit D). Encourage weight-bearing activity & physiotherapy. Minimise cumulative GC dose (use GC-sparing regimens). Vitamin D & calcium supplementation as needed. Incorporation of Bisphosphonates if presence of severe osteoporosis or fractures. Monitoring of BP, fasting glucose, lipid profile and disproportionate limb fat loss and central gain (lipodystrophy red flag). Educate families on bone & metabolic health risks.
